# Supplementary material for: Complexome profiling of the Chlamydomonas psb28 mutant reveals TEF5 as an early PSII assembly factor
Source: Plant Cell. 2025 Mar 17;37(6):koaf055. doi: 10.1093/plcell/koaf055 (PMC12164590; doi:10.1093/plcell/koaf055)
Supplement: koaf055_Supplementary_Data [file koaf055_supplementary_data.zip › psb28_interactive.zip!/plots/intensity/qualityPlots/peptides/NUOAF4_Cre06.g274650.t1.1.html]

### Cre06.g274650.t1.1

Trivial name: NUOAF4  
SeqCoverage [median]: 31.60  
Mass: 67.99 kDa  
  
 

| Sequence | MissedCleavage | Mass [Da] | wtIntensity | muIntensity | Score |
| --- | --- | --- | --- | --- | --- |
| AQIVAQVAVIR | 0 | 1166.71 | 0.00 | 8230.08 | 135.79 |
| AQSDPEGVPLDPSR | 0 | 1466.70 | 9320.18 | 10688.61 | 174.72 |
| ARDVLLGSGEGK | 1 | 1200.65 | 744.22 | 937.93 | 58.32 |
| AVPPPPPVTDER | 0 | 1273.67 | 655.54 | 1188.80 | 42.74 |
| AVPPPPPVTDERR | 1 | 1429.77 | 4773.69 | 8773.58 | 90.43 |
| DVLLGSGEGK | 0 | 973.51 | 7709.55 | 17891.15 | 115.71 |
| EKVETKEPVGANA | 2 | 1370.70 | 6112.13 | 10867.62 | 122.46 |
| ENAVLVLGATGR | 0 | 1198.67 | 5806.19 | 22290.24 | 193.28 |
| LDDVIMGGASDSGLEPISEAER | 0 | 2260.05 | 0.00 | 547.93 | 124.45 |
| LGLDLSGSDGIHMR | 0 | 1469.73 | 1093.18 | 25707.61 | 77.91 |
| LGLVLSR | 0 | 756.49 | 2122.71 | 21629.24 | 94.41 |
| MGLTEGR | 0 | 762.37 | 2955.63 | 8171.18 | 163.84 |
| MQDIPIVQLNPNGTLNHK | 0 | 2031.06 | 0.00 | 12166.26 | 111.58 |
| NAIIGDDEVKR | 1 | 1228.64 | 12254.68 | 28015.74 | 134.75 |
| RAQIVAQVAVIR | 1 | 1322.81 | 0.00 | 5924.02 | 150.10 |
| RAQSDPEGVPLDPSR | 1 | 1622.80 | 1109.45 | 2812.38 | 55.19 |
| SSGYPYTVVR | 0 | 1127.56 | 10681.89 | 25645.05 | 148.52 |
| TAEDLAVWNK | 0 | 1145.57 | 280.94 | 1466.41 | 141.20 |
| VETKEPVGANA | 1 | 1113.57 | 5533.64 | 16932.98 | 226.20 |
| VGLQPMPK | 0 | 868.48 | 7267.92 | 14159.62 | 128.75 |
| VLGDGQTFK | 0 | 963.50 | 8729.45 | 15662.14 | 92.44 |
| VLGDGQTFKMNIK | 1 | 1449.77 | 505.13 | 3627.41 | 47.29 |
| YTAEIAVR | 0 | 921.49 | 2072.79 | 12579.34 | 157.58 |
|  |  |  | 89728.9 | 275915.3 |  |
